# Maternal childhood trauma is associated with offspring body size during the first year of life

**DOI:** 10.1038/s41598-022-23740-6

**Published:** 2022-11-15

**Authors:** Anna Apanasewicz, Dariusz P. Danel, Magdalena Piosek, Patrycja Wychowaniec, Magdalena Babiszewska-Aksamit, Anna Ziomkiewicz

**Affiliations:** 1 Department of Anthropology, Hirszfeld Institute of Immunology and Experimental Therapy, Polish Academy of Sciences, 12 Weigla Street, 53-114 Wrocław, Poland; 2 Institute of Psychology, University of Wroclaw, 1 Dawida Street, 50-527 Wrocław, Poland; 3 Department of Human Biology, University of Wroclaw, 63 Przybyszewskiego Street, 51-148 Wroclaw, Poland; 4 Laboratory of Anthropology, Institute of Zoology and Biomedical Research, Jagiellonian University, Gronostajowa 9, 30-387 Kraków, Poland

**Keywords:** Evolutionary developmental biology, Epidemiology

## Abstract

Maternal childhood trauma (MCT) is an important factor affecting offspring size at birth. Whether the effect of MCT persists during the subsequent development remains unclear. We present the results of a semi-longitudinal investigation examining the physical growth of infants born to mothers with high (HCT) and low (LCT) childhood trauma during the first year of life. One hundred healthy mother-infant dyads were included based on following criteria: exclusive breastfeeding, birth on term with appropriate weight for gestational age. MCT was assessed using the Early Life Stress Questionnaire. The weight, length, and head circumference of the infant were taken at birth, 5 and 12 months postpartum. Separate MANCOVA models were run for infant size at each age. We found an association between MCT and infant size at 5 and 12 months. The children of mothers with HCT had higher weight and greater head circumference than the children of mothers with LCT. These results suggest that MCT might contribute to developmental programming of offspring growth during the first year of life. From an evolutionary perspective, the larger size of HCT mother's offspring might represent an adaptation to potentially harsh environmental conditions. This effect might be mediated by epigenetic changes to DNA and altered breast milk composition.

## Introduction

The prenatal period is a crucial stage of individual development with long-term consequences for subsequent well-being and health^1–3^. This period is characterized by increased vulnerability and sensitivity to environmental disruptors that, if persistent, can disrupt prenatal development and result in fetal growth retardation^4^. One of such factors that influence pre- and postnatal development is maternal stress^5^.

Studies have shown that the placental mechanisms can protect the fetus against high maternal stress to some extent; however, these mechanisms are insufficient to completely diminish the effect of increased stress^6^. For example, the placental enzyme 11β-hydroxysteroid dehydrogenase, which converts cortisol to its less physiologically active form cortisone^7,8^, only does so to a certain extent; therefore, maternal and fetal cortisol levels during high stress periods correlate with each other^8^. The observed effects of maternal stress on the development of offspring include increased reactivity of the hypothalamus–pituitary–adrenal (HPA) axis ^9,10^, changes in body size and composition at birth and during postnatal life^11,12^. More specifically, a high level of maternal stress during pregnancy has been found to be associated with a lower weight, length, and head circumference at birth in several studies^11–14^, however, some authors have shown a positive or nonsignificant effect^15–17^.

Interestingly, a new body of evidence suggests that some maternal stressors experienced even before pregnancy might influence the growth and well-being of an offspring^18^. In particular, extreme traumatic stress during childhood can affect the sensitivity of the HPA axis and, consequently, lead to its long-term dysregulation^19,20^. For example, pregnant women who experience traumatic stress during childhood have a higher awaking cortisol response^21,22^. Recent studies investigating the effects of maternal childhood trauma (MCT) suggest that these developmental effects may be transgenerational (transmitted to the offspring). Although most studies have focused on the behavioral and emotional consequences of MCT, some of them suggest that the biological development of the offspring might be also affected^23–25^. For example, MCT, including violence, is associated with preterm birth^26^, lower birth weight^24,27^ (although this effect is not universal across all studies^28^), lower infant intracranial volume^25^, and a higher cephalization index^23^.

Our preliminary results showed that body weight and head circumference are positively associated with increased maternal childhood trauma among exclusively breastfed infants^29^. Simultaneously, the study also suggested that neither catch-up growth nor breast milk energy density contributed to the observed growth effects^29^. While MCT-associated changes in infant body size have been detected in the very early stages of development–at birth and early postpartum, it remains unclear whether these changes persist in later stages of development. To address this, we studied body size in relation to the MCT level in the same breastfed infants during the first year of life. We hypothesized that the differences in the growth pattern between infants of mothers with high childhood trauma (HCT) and low childhood trauma (LCT) observed during the initial months would persist further in their development.

## Materials and methods

### Study group and protocol

The study group consisted of 100 mother-infant dyads from Poland. The recruitment of dyads took place when the babies were about 5 months old and was based on the following inclusion criteria: (a) for mothers: age older than 18 years old; not taking steroid medication, smoking, or drinking alcohol during pregnancy or lactation; without metabolic or congenital diseases (b) for infants: being born from a single and uncomplicated pregnancy; at least 37 weeks of gestation with birth weight at least 2600 g and exclusively breastfed for at least 5 months. The above criteria where established based on the literature indicating that children born prematurely or small for gestational age have a different developmental pattern^30–32^, especially during the first year of life. Most of them experience catch-up growth, but some remain consequently smaller^33^ during this period. Thus, including prematurely born infants in the study group could potentially confound the results.

The study protocol included two meetings with mothers and infants. The first meeting occurred when the babies were approximately 5 months old. During this meeting, we collected maternal and infant measurements, information on maternal socioeconomic status and life satisfaction, birth outcomes, and postpartum depression. At the second meeting that took place when the children were about 12 months, maternal and infant measurements were collected again. At this point, mothers were also asked about their traumatic experience during childhood. This research protocol was approved by the Bioethical Committee of Lower Silesian Medical Chamber in Wroclaw (protocol code 1/NT/2016 from 10.02.2016).

Each participant in the study received information about the course and purpose of the study, giving informed, written consent to participate in the study in accordance with the tenets of the Declaration of Helsinki. Respondents were allowed to opt out of the study at any stage of the study, and there were no legal or financial consequences for opting out.

### Maternal childhood trauma, postpartum depression, and socioeconomic status

The MCT was evaluated using the Polish version of the Early Life Stress Questionnaire (ELSQ)^34^, which is an international psychological tool constructed based on the Child Abuse and Trauma Scale^35^. Women were asked to indicate their childhood traumatic events up to the age of 12 years. The questionnaire included 19 events such as peer bullying, domestic violence, sexual harassment, long-term illness, or natural disasters. Each event scored 1 point.

Experiencing symptoms of maternal postnatal depression (maternal PD risk) was evaluated using the Polish version of the Edinburgh Postpartum Depression Scale (EPDS). This 4-point and 10-item questionnaire is a widely used tool in clinical and nonclinical settings^28,36^. Following other studies, including those conducted on Polish samples, the cut-off point for higher risk of postpartum depression was defined by a score value of at least 14^36^. Additionally, the participants were asked to assess their financial satisfaction on a 7-point Likert scale (1- very unsatisfied; 7- very satisfied) and declare their educational status (higher education with at least Bachelor's or nonhigher education).

### Anthropometric measurements

Anthropometric measurements of the dyads were taken twice. First, when the infants were approximately five months old and second, at twelve months. Maternal body weight was measured with a Tanita SC-240 MA scale (accuracy of 0.1 kg) and height with a stadiometer (accuracy of 0.1 cm). The mothers also reported their pre-pregnancy body weight. The measurements were used to calculate body mass index (BMI; BMI = body weight [kg]/body height [cm]^2). For infants, measurements included body length using a Seca measuring board (model 417; accuracy of 0.1 cm), weight using an analog hospital scale (with an accuracy of 0.1 kg), and head circumference using a measuring tape (with the accuracy of 0.1 cm). Birth outcomes (gestational age, weight, length, and head circumference) were taken from the child's health record.

### Statistical methods

Mothers were divided into LCT or HCT groups according to the median value (Me = 2) of the ELSQ score. Differences in mean values of main study variables between LCT and HCT mothers were tested using t-test. Differences in the number of mothers according to increased depression risk and infant sex between LCT and HCT group was tested using chi^2^ test. The association between infant weight, length, head circumference, and maternal childhood trauma was tested using General Linear Models. The separate multivariate analyses of covariance (MANCOVA) models were built for growth parameters at each infant age (at birth, 5 and 12 months) with all size parameters as dependent variables, the level of MCT (low–high), infant sex (boy-girl) and risk of maternal postpartum depression (low–high) as categorical predictors, and maternal BMI and infant age as covariates. Following the MANCOVA models, we also ran separate univariate analyses of covariance (ANCOVA) to test which of the dependent variables were statistically significant. In addition, Cohens’ d values (group comparisons with different sample size)^37^ was calculated to quantify the effect of MCT on infant body size parameters.

The Henze-Zirkler test indicated multivariate normality of the dependent variables, and the Box M test confirmed the homogeneity of the variance–covariance matrices. Since the deviations from normality were relatively minor and our sample size was sufficient to obtain robust results (n > 30 in both groups), we followed the standard parametric procedure for the univariate analysis. Statistical analysis was performed using StatSoft STATISTICA (data analysis software system), version 12 (www.statsoft.com), and the R statistical environment (version 3.6.0). The statistical significance level was established at *p* < 0.05, however, we also reported a marginally significant effect at 0.06 > *p* > 0.05.

## Results

The ELSQ score in the study group ranged from 0 to 11 traumatic events during childhood. 46% (n = 46) of the participants suffered from more than 2 traumatic events up to 12 years of age, while only 14% (n = 14) of the women in the study group did not experience any traumatic events. Therefore, mothers who experienced more than 2 traumas were included in the HCT group. Out of all participating mothers, 14 (14%) had an increased risk of postpartum depression according to the defined cut-off point when their babies were five months old. The results of the chi^2^ test indicated that there was no association between the MCT and the risk of postpartum depression. The number of women with an increased risk of postpartum depression did not differ between the LCT and HCT groups (chi^2^ < 0.01, *p* = 0.972). Furthermore, neither maternal economic satisfaction nor education was associated with MCT (Table 1).

**Table 1:** Maternal and infant characteristics in all, HCT and low LCT participants at child’s birth, at 5, and 12 months. Significant differences asterisked.

Birth outcomes				
	All participants (N = 95) Mean (SD)	HCT (N = 44) Mean (SD)	LCT (N = 51) Mean (SD)	*p*
ELSQ score	2.73 (2.28)	4.76 (1.88)	1.13 (0.83)	< 0.001*
Maternal age [years]	30.47 (3.82)	29.65 (3.48)	31.18 (3.99)	0.051
Maternal BMI before pregnancy [kg/m^2]	22.73 (3.50)	23.22 (4.06)	22.31 (2.92)	0.211
Infant sex [boys%]	55.79	59.09	52.94	0.547
Gestational age [weeks]	39.92 (1.41)	40.09 (1.51)	39.77 (1.32)	0.264
Infant body length [cm]	54.74 (2.90)	55.07 (2.82)	54.45 (2.96)	0.302
Infant weight [g]	3,506.37 (443.69)	3,569.77 (475.51)	3,451.67 (411.16)	0.197
Infant head circumference [cm]	34.02 (1.72)	34.02 (2.03)	34.02 (1.42)	0.993

Infant age approximately 5 months				
	All participants (N = 100) Mean (SD)	HCT (N = 46) Mean (SD)	LCT (N = 54) Mean (SD)	*p*
Financial satisfaction [7-points scale]	5.49(1.03)	5.54(1.09)	5.44(0.98)	0.676
Education [high %]	94.00	91.30	96.30	0.910
ELSQ score	2.81 (2.32)	4.86 (1.90)	1.16 (0.83)	< 0.001*
Maternal age [years]	31.19 (4.04)	30.13 (3.42)	31.98 (4.36)	0.022*
Maternal BMI [kg/m^2]	23.04 (3.56)	23.49 (4.00)	22.67 (3.13)	0.253
Infant sex [boys %]	57.00	58.70	55.56	0.295
Infant age [months]	4.76 (0.58)	4.71 (0.54)	4.81 (0.61)	0.415
Infant body length [cm]	66.12 (3.04)	66.38 (3.25)	65.89 (2.86)	0.422
Infant weight [g]	7,101.53 (920.02)	7,350.46 (1,001.32)	6,878.13 (786.62)	0.008*
Infant head circumference [cm]	42.08 (1.44)	42.40 (1.50)	41.81 (1.35)	0.044*

Infant age approximately 12 months				
	All participants (N = 95) Mean (SD)	HTC (N = 43) Mean (SD)	LTC (N = 52) Mean (SD)	*p*
ELSQ score	2.81 (2.32)	4.86 (1.90)	1.16 (0.83)	< 0.001*
Maternal age [years]	31.77 (3.62)	31.09 (3.11)	32.32 (3.93)	0.098
Maternal BMI [kg/m^2]	22.15 (3.81)	22.71 (4.36)	21.68 (3.26)	0.191
Infant sex [boys%]	57.89	60.47	55.77	0.645
Infant age [months]	12.36 (0.73)	12.35 (0.84)	12.36 (0.63)	0.972
Infant body length [cm]	76.12 (3.23)	76.76 (3.71)	75.60 (2.70)	0.080
Infant weight [g]	9,514.63 (1,035.09)	9,841.86 (1,025.39)	9,244.04 (971.57)	0.005*
Infant head circumference [cm]	46.00 (1.64)	46.40 (1.50)	45.68 (1.68)	0.028*

Significant differences between groups in the infant size characteristics (body weight, and head circumference) were found at the age of 5 and 12 months (Table 1). In particular infants of HCT mothers were significantly heavier (t = −2.71 *p* = 0.008; t = −2.91, *p* = 0.005 at 5 and 12 months respectively) and had larger head circumference than infants of LCT mothers (t = −2.04, *p* = 0.044; t = −2.27, *p* = 0.028 for 5 and 12 months, respectively). No significant differences were found in infant body size at birth and the gestational age. Maternal characteristics also did not differ between the HCT and LCT groups, excluding maternal age when the children were 5 months old (Table 1). This accidental but significant difference in age had no effect on any of the infant size parameters.

The MANCOVA models show, that the differences between LCT and HCT in infant growth characteristics (Wilks λ = 0.90, F _(3,92)_ = 3.57, *p* = 0.0 17) remained significant at the age of 5 months and marginally significant at the age of 12 months (Wilks λ = 0.92, F _(3,87)_ = 2.61, *p* = 0.056) after controlling for maternal BMI and risk of postpartum depression as well as infant sex, and age (Table 2). The effect of MCT on growth parameters at birth was not statistically significant (Wilks λ = 0.98, F _(3,87)_ = 0.64, *p* = 0.592).

**Table 2:** Results of MANCOVA models for the association between level of maternal childhood trauma (LCT, HCT), maternal BMI, maternal postnatal depression risk infant age, sex, and child growth parameters at birth, at 5, and 12 months. Significant effects asterisked and marginally significant underlined.

Model	Wilks λ	F	ηp^2^	*p*
**Growth parameters at birth**				
Intercept	0.46	33.86	0.54	< 0.001*
Maternal childhood trauma (LCT-HCT)	0.98	0.64	0.02	0.592
Maternal BMI before pregnancy	0.86	4.91	0.14	0.003*
Maternal PD risk (low–high)	0.99	6.38	0.01	0.187
Gestational age	0.82	6.38	0.18	0.001*
Infant sex	0.94	1.94	0.06	0.129
**Growth parameters at 5 months**				
Intercept	0.09	313.77	0.91	< 0.001*
Maternal childhood trauma (LCT-HCT)	0.90	3.57	0.10	0.017*
Maternal BMI	0.90	3.56	0.10	0.017*
Maternal PD risk (low–high)	0.99	0.42	0.01	0.736
Infant age	0.78	8.67	0.22	< 0.001*
Infant sex	0.77	9.02	0.23	< 0.001*
**Growth parameters at 12 months**				
Intercept	0.19	122.89	0.81	< 0.001*
Maternal childhood trauma (LCT-HCT)	0.92	2.61	0.08	0.056
Maternal BMI	0.84	5.42	0.16	0.002*
Maternal PD risk (low–high)	0.93	2.19	0.07	0.095
Infant age	0.72	11.17	0.28	< 0.001*
Infant sex	0.70	12.16	0.30	< 0.001*

**Figure 1:**
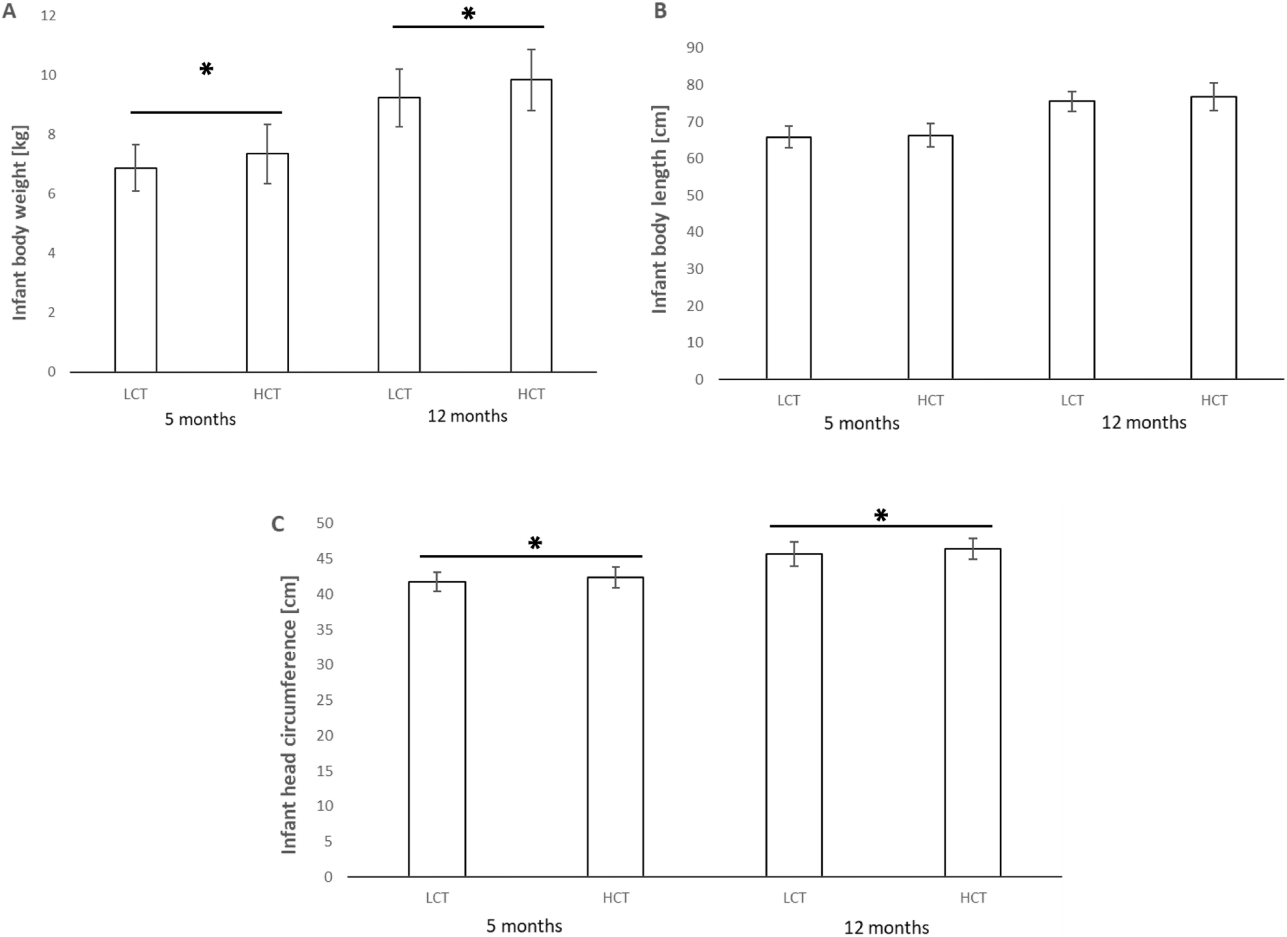
Mean and standard deviations for infant body weight (**A**), length (**B**) and head circumference (**C**) in LCT and HCT mothers at the age of 5, and 12 months. Significant differences asterisked.

The results of the univariate analysis did not show a significant effect of maternal trauma at birth (Table3). In contrast, a significant association between the level of MCT and infant weight (F _(1,94)_ = 8.06, *p* = 0.006, Cohen’s d = 0.53) as well as head circumference (F _(1,94)_ = 6.17, *p* = 0.015, Cohen’s d = 0.42) was found at 5 months (Table 4 and Fig. 1). Similar effects were observed for both also at 12 months (F _(1,89)_ = 7.17, *p* = 0.009, d = 0.60; F _(1,89)_ = 5.06, *p* = 0.027, d = 0.45 for weight and head circumference, respectively) (Table 5 and Fig. 1). The effect of MCT on body length at 5 and 12 months was not significant.

**Table 3:** Results of univariate analysis of MANCOVA models for birth outcomes. Significant effects asterisked.

Model	F (1,89)	*p*
**Body length**		
Intercept	7.57	0.007*
Maternal childhood trauma (LCT-HCT)	0.04	0.847
Maternal BMI before pregnancy	10.43	0.002*
Maternal PD risk (low–high)	0.15	0.697
Gestational age	13.54	0.004*
Infant sex	4.42	0.038*
**Body weight**		
Intercept	3.89	0.052
Maternal childhood trauma (LCT-HCT)	0.15	0.696
Maternal BMI before pregnancy	13.56	< 0.001*
Maternal PD risk (low–high)	0.00	0.993
Gestational age	18.45	< 0.001*
Infant sex	4.45	0.038*
**Head circumference**		
Intercept	15.16	< 0.001*
Maternal childhood trauma (LCT-HCT)	0.53	0.470
Maternal BMI before pregnancy	7.31	0.001*
Maternal PD risk (low–high)	0.27	0.60
Gestational age	6.47	0.013*
Infant sex	3.20	0.077

**Table 4:** Results of univariate analysis of MANCOVA models for body parameters at the age of 5 months. Significant effects asterisked.

Model	F (1, 94)	*p*
**Body length**		
Intercept	293.36	< 0.001*
Maternal childhood trauma (LCT-HCT)	0.69	0.408
Maternal BMI	1.32	0.254
Maternal PD risk (low–high)	0.86	0.355
Infant age	4.77	0.032*
Infant sex	5.35	0.023*
**Body weight**		
Intercept	13.24	< 0.001*
Maternal childhood trauma (LCT-HCT)	8.06	0.006*
Maternal BMI	8.37	0.005*
Maternal PD risk (low–high)	0.44	0.511
Infant age	10.16	0.002*
Infant sex	12.51	< 0.001*
**Head circumference**		
Intercept	698.87	< 0.001*
Maternal childhood trauma (LCT-HCT)	6.17	0.015*
Maternal BMI	6.84	0.010*
Maternal PD risk (low–high)	1.00	0.320
Infant age	25.86	< 0.001*
Infant sex	26.16	< 0.001*

**Table 5:** Results of univariate analysis of MANCOVA models for body parameters at the age of 12 months. Significant effects asterisked.

Model	F (1,89)	p
**Body length**		
Intercept	79.63	< 0.001*
Maternal childhood trauma (LCT-HCT)	3.09	0.082
Maternal BMI	3.21	0.077
Maternal PD risk (low–high)	5.62	0.020*
Infant age	29.48	< 0.001*
Infant sex	8.73	0.004*
**Body weight**		
Intercept	0.54	0.466
Maternal childhood trauma (LCT-HCT)	7.17	0.009*
Maternal BMI	15.475	< 0.001*
Maternal PD risk (low–high)	2.88	0.093
Infant age	15.08	< 0.001*
Infant sex	6.38	0.013*
**Head circumference**		
Intercept	193.36	< 0.001*
Maternal childhood trauma (LCT-HCT)	5.06	0.027*
Maternal BMI	2.23	0.139
Maternal PD risk (low–high)	0.08	0.775
Infant age	14.40	< 0.001*
Infant sex	32.71	< 0.001*

## Discussion

The current semi-longitudinal study for the first time demonstrates that although maternal childhood trauma does not affect infant size at birth, it is significantly and positively associated with infant size during the first year of life. Mothers with HCT had infants with almost 10% higher weight and 2% greater head circumference than mothers with LCT at the age of 5 and 12 months.

The presented results corroborate the results of an experimental study in hens showing that traumatic stress experienced during early life and puberty is related to earlier hatching of offspring and their increased body weight at the age of one month^38^. In contrast, a human study by Choi et al. ^28^ has found a negative but indirect association between maternal childhood trauma and infant body size (weight and length) via maternal postpartum depression. Almost 30% of the participants in this study suffered from postpartum depression, which in turn predicted a negative infant development outcome at the age of 12 months^28^. The low prevalence of postpartum depression observed in our study (about 14% of the participants) could be related to a common practice of universal and exclusive breastfeeding among the study participants, which has been previously shown to have beneficial effects on maternal well-being^39^ and infant development^40^. Contrasting findings also came from the study by Smith et al.^24^, who reported a lower birth weight in infants born to mothers who experienced HCT than those with LCT. The discrepancies in the findings between this and our study could be due to the fact that the former did not control for preterm birth and/or low gestational age, both associated with decreased weight at birth^24^.

The higher weight in babies born to HCT mothers observed in our study might be associated with impaired glucose metabolism^41,42^ which in the future might result in an increased risk of glucose intolerance and metabolic syndrome. Such effects usually co-occur with overweight and obesity^2,43^ and have been previously reported in adult descendants of parents with higher trauma^44^. In addition, higher body weight during infancy is related to the risk of being overweight and obesity during adulthood^45^. Thus, the results of our study suggest that the tendency towards an increased weight among children born to HCT mothers might be long-lasting, and probably result in a higher risk of metabolic conditions in adulthood.

Our analysis also showed a larger head circumference among infants of HCT mothers compared to the infant of LCT mothers. This result is in line with a study by Appleton et al.^23^, who found that infants born to women with HCT had a higher cephalization index. An increase in the value of this index is usually associated with an increase in head circumference.

Overall, these results suggest that MCT experience may induce intergenerational changes in physical development even without trauma present in the next generation^46^. These effects are hypothesized to be mediated by epigenetic effects on germline and somatic cells, including DNA methylation and histone and RNA modifications^46^. Breast milk, which contains noncoding RNAs, such as microRNAs, serves as epigenetic vectors in molecular communication between mother and offspring and constitutes the first vital gate allowing these developmental effects^46^. Furthermore, HCT may program HPA axis reactivity to produce an increased level of stress hormones, including cortisol, many years after exposition^47^. Thus, cortisol transmitted from serum to breast milk might serve as a second gate^48^. Glucocorticoid levels during the perinatal period demonstrate a long-term programming effect on growth and health during later life^48,49^. Recent literature has shown, that higher values of head circumference and body weight are related to altered levels of fatty acids^50,51^ and glucocorticoids^52–54^ in breast milk. Additionally, our previous research showed that maternal stress reactivity is positively associated with the level of polyunsaturated fatty acids in milk^55^, which are crucial for brain growth and development^56^. It is important to note that the infants in our study group were breastfed exclusively for at least 5 months. Thus, it is possible that maternal HCT was reflected in a modified level of polyunsaturated fatty acids and higher cortisol in milk and, as a result, a faster increase in body mass and head circumference.

From the evolutionary perspective, a larger size of offspring born to HCT mothers might result from a faster life pace, as posited by the Life History Theory. The term Life History was introduced by Stearns in 1992 and emphasizes that environmental conditions can push individuals into two types of life strategies: fast and slow^57^. Individuals that exist under harsh environmental conditions, higher levels of stress and increased risk of mortality must adapt, so such conditions would result in accelerated sexual maturation (e.g., early rapid fat gain) and earlier successful reproduction^58^. On the other hand, it would be associated with significant costs to health and longevity^58,59^. For example, early life adiposity predisposes to obesity and other metabolic disorders in adulthood, while earlier and faster reproduction generates considerable metabolic cost to the individual^60^. According to this theory we postulate that the MCT can be, to some extent, treated as an indicator of a stressful environment and harsh condition within the meaning of the Life History Theory. It is also possible that the observed effect may represent a mismatch between maternal and offspring developmental environments, where the maternal adaptive response to a harsh environment might induce long-term changes in offspring development, even in the absence of ecological obstacles.

One of the limitations of our study might be the fact that participants childhood traumatic events retrospectively. However, this limitation is difficult to avoid due to the extended period between maternal childhood and pregnancy. We also did not know how often and for how long women had been exposed to the reported experiences. To minimize the recall bias, we assessed MCT using a standardized psychological questionnaire, which was successfully applied in several other studies^34,61–63^. Furthermore, we did not control for post-traumatic stress disorder (PTSD) and resilience as the additional factors in the analysis. Several studies have underlined the effect of childhood trauma on the prevalence of PTSD^64^. The latter has been shown to have a long-lasting effect on maternal metabolism and as a consequence, on the development and health of the offspring^65^. Whereas resilience is postulated as a protective factor with the potential to decrease the negative effect of childhood trauma on the development of offspring and future health^66^. Finally, the information about birth outcomes was collected from child health records instead of being measured, so the measurement protocol could not have been entirely consistent between different hospitals.

Our study demonstrates that MCT is significantly associated with the size of the offspring during the first year of life even after adjusting for other significant factors that influenced body size such as maternal BMI and postnatal depression, infant age, sex, and age. Children of mothers with HCT had higher weight and larger head circumference than peers born to mothers with LCT, and this effect was independent of body size at birth. These results suggest that MCT might contribute to alterations in the maternal physiology of the HPA axis, which in turn program the development of offspring in the long-term perspective. We propose that this effect may arise from the faster pace of life syndrome mediated by epigenetic changes to DNA and the altered composition of breast milk.

## Supplementary Information

Supplementary Information.

## Data Availability

The data set analyzed during the current study is available from the corresponding author on request.
